# Complications of atypical pneumonia: A case of *Legionella longbeachae* empyema

**DOI:** 10.1002/rcr2.1281

**Published:** 2024-02-01

**Authors:** Amy Samson, Michael J. Maze

**Affiliations:** ^1^ Respiratory Department Te Whatu Ora Waitaha Canterbury Christchurch New Zealand; ^2^ Department of Medicine University of Otago Christchurch Christchurch New Zealand

**Keywords:** empyema *Legionella longbeachae*, Legionnaires' disease, lung abscess, pleural effusion

## Abstract

*Legionella longbeachae* is the most common cause of Legionnaires' disease in Australasia. *Legionella* species are considered a rare cause of pleural infection, and empyema and lung abscess due to *L. longbeachae* has not previously been reported. Our patient presented with a 2–3 week history of breathlessness, lethargy, dry cough and headaches. Initial chest radiograph showed extensive left sided consolidation with an associated pleural effusion. An area of necrotising pneumonia evident on computed tomography scan evolved into a multiloculated intrapulmonary abscess. Sputum culture isolated *L. longbeachae,* which prompted culture of pleural fluid on buffered charcoal yeast extract agar and isolation of the organism. This case provides evidence that *L. longbeachae* can cause both empyema and lung abscess, and in areas where it is prevalent, increased use of *Legionella* specific agar for pleural fluid culture should be considered.

## Graphical abstract

*Legionella longbeachae* is a common cause of pneumonia in Australasia but rarely identified as the cause of empyema. We report a case of *L. longbeachae* empyema and lung abscess and highlight the need for specific culture media or molecular testing to identify this organism in pleural fluid.

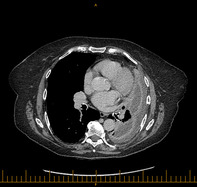

## INTRODUCTION

First described in 1977, the leading cause of Legionnaires' disease globally is *Legionella pneumophila*, but in Australasia *Legionella longbeachae* predominates. Legionella pneumonia is most common in spring and summer, associated with gardening and exposure to soil or compost.^1^ Parapneumonic effusions are often identified on chest radiographs in patients with *L. longbeachae* pneumonia.^2^ However, previous research suggests that atypical pathogens, including *Legionella* species are seldom the cause of empyema.^3^ Empyema is a life threatening infection of the pleural space requiring pleural drainage and targeted antimicrobial treatment. *L. longbeachae* empyema has not previously been reported but may be underreported as diagnosis requires culture of *Legionella* species using specific media or molecular testing, which are rarely performed on pleural fluid.

## CASE REPORT

**FIGURE 1:**
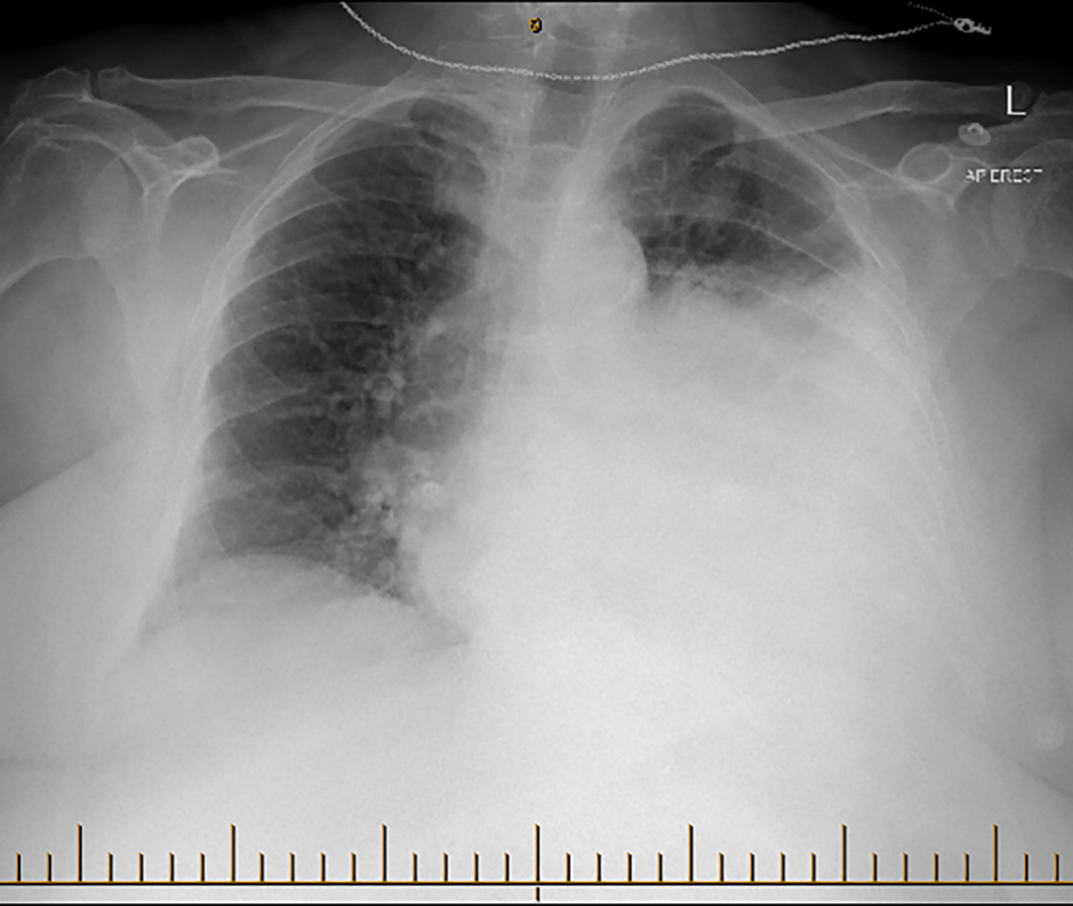
Chest radiograph on day of admission.

A 77 year old woman with a history of asthma presented with a 2–3 week history of dyspnoea, lethargy and dry cough, with a deterioration in her symptoms a few days prior to admission. In the community, she was prescribed three courses of amoxicillin and one course of prednisone, with minimal improvement. On admission, she was noted to have raised inflammatory markers with a white cell count of 31.5 × 10^9^/L, C‐reactive protein of >480 mg/L and an acute kidney injury with a creatinine of 127 umol/L. A chest radiograph showed extensive left sided consolidation with a likely associated pleural effusion (Figure 1).

A keen gardener, it was noted that just prior to the onset of illness she had used commercially produced bagged compost, without mask protection. She was treated with intravenous amoxicillin‐clavulanate and clarithromycin. Following evidence of a moderate left sided pleural effusion on pleural ultrasonography, a chest drain was inserted, initially draining 310 mL of pleural fluid. Despite fluid resuscitation and antibiotic escalation to piperacillin‐tazobactam, the patient was persistently hypotensive and oliguric, and required vasopressor support.

Laboratory analysis of the pleural fluid showed a pH of 7.12, protein of 36 g/L, glucose 2.9 mmol/L and lactate dehydrogenase 3116 U/L, consistent with an exudate and likely empyema in the clinical context. Further analysis showed a pleural fluid white cell count of 12,400 × 10^6^, predominantly consistent of polynucleated leucocytes, with no organisms seen on gram stain. Polymerase chain reaction (PCR) testing of sputum detected *L. longbeachae* which was subsequently confirmed by culture. Having isolated *L. longbeachae* in the sputum, Buffered Charcoal Yeast Extract (BCYE) Agar was used to culture the pleural fluid, which also isolated *L. longbeachae*.

**FIGURE 2:**
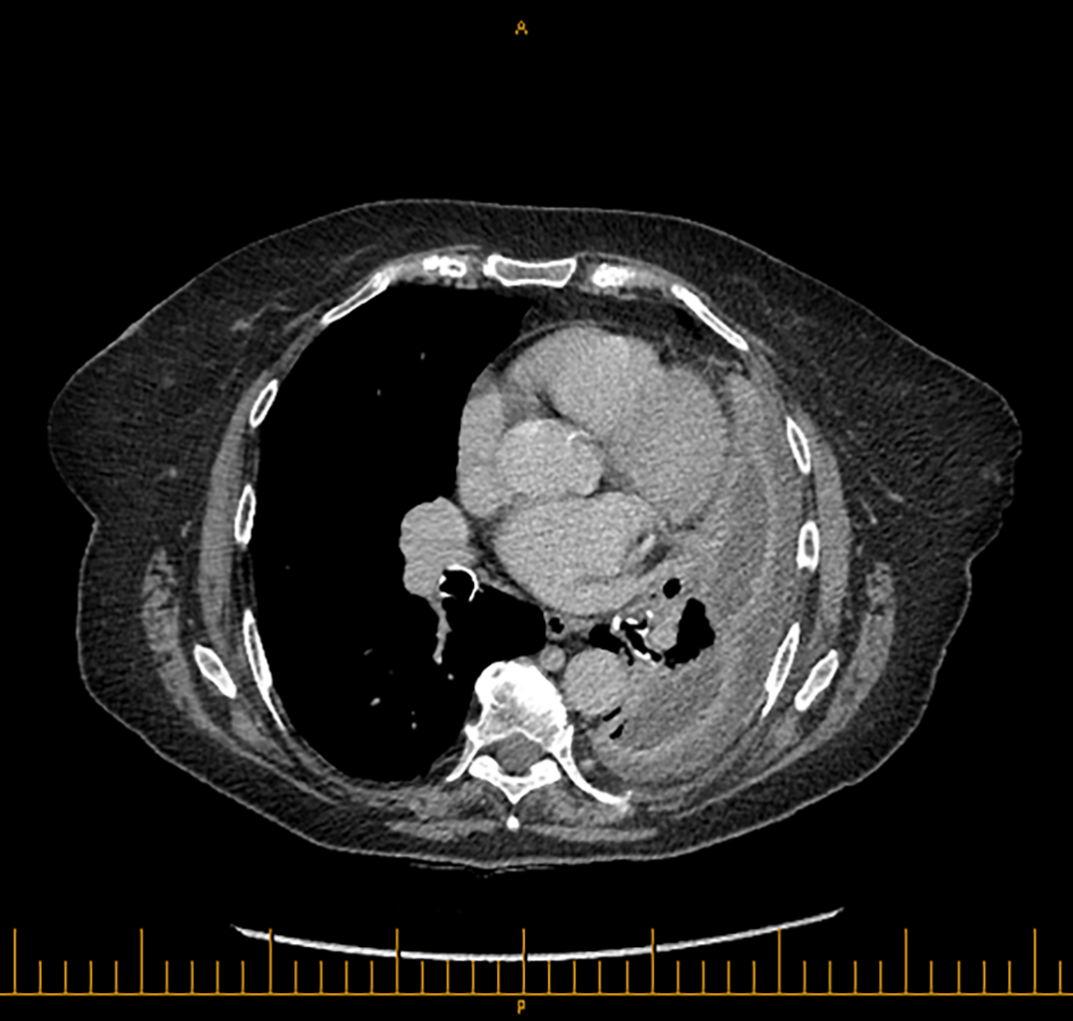
Axial slice of chest computed tomography scan taken 2 months following admission.

The patient was transitioned to oral amoxicillin and azithromycin and treated with a total of 4 weeks of antibiotics. However, she re‐presented within 2 weeks of completing treatment with worsening cough. Subsequent chest radiograph and computed tomography scan confirmed development of a multiloculated abscess cavity within the left lower lobe (Figure 2). Sputum cultured oropharyngeal flora and culture on BCYE agar was negative; but PCR testing of the sputum detected *L. longbeachae* on three further occasions, up to 17 weeks following initial presentation. She was restarted on oral amoxicillin‐clavulanate, and initially roxithromycin and subsequently ciprofloxacin for a further 3 months. Following completion of treatment her cough and fever resolved, and her chest radiograph showed resolution of the abscess with linear scarring.

## DISCUSSION

*Legionella* species have rarely been found to be the cause of empyema. However, our case provides evidence that *L. longbeachae* is capable of causing both lung abscess and empyema. This adds to existing case reports of lung abscess and empyema caused by *Legionella* species such as *L. pneumophila and L. micdadei*. This highlights the importance of testing for *Legionella* species and considering them as a cause of empyema and lung abscess, in order to facilitate prompt targeted antimicrobial treatment.

In a study of 374 pleural fluid samples in patients with parapneumonic effusions, Wrightson et al. detected pathogens in 89%, through amplification of the 16S gene with nested PCR assays. Assays detected just two (0.5%) cases of *Mycoplasma* and no cases of *L. pneumophila*. Consequently, the authors and subsequent guidelines concluded that empirical treatment of pneumonia with pleural effusions did not require antibiotic cover for atypical organisms.^3^, ^4^ However, the use of primers specific for *L. pneumophila* limits generalisability of this result to regions with a high incidence of *L. longbeachae* infection.

Although *Legionella* species are considered a rare cause of empyema, a large case series of Legionnaires' disease, caused predominantly by *L. longbeachae*, found 22% had an associated pleural effusion.^2^ Detection of *Legionella* species in clinical specimens is challenging as culture requires special media and has low sensitivity. Molecular methods are more sensitive,^5^ but are seldom used. As such, testing for *Legionella* species is rarely performed and consequently the prevalence of *L. longbeachae* empyema may be underestimated. Detection of *Legionella* species is essential as Legionnaires' disease is associated with a high morbidity and lack of specific antimicrobial treatment is associated with worse outcomes.^2^

The development of a lung abscess is likely a consequence of necrotic lung tissue relating to the Legionnaires' disease. The molecular detection, but inability to culture *L. longbeachae* in sputum later in the illness may indicate secondary infection with other bacterial species playing a role in abscess formation.

Our case provides evidence that *Legionella longbeachae* infection can cause lung abscess and empyema. As a result, specific testing should be considered in patients at epidemiological risk. In countries where *L. longbeachae* is a common cause of pneumonia, systematic testing of pleural fluid for *Legionella* species is needed to better understand the prevalence of *Legionella* empyema. Furthermore, when considering empirical antibiotic treatment, the presence of a pleural effusion in patients with pneumonia should not discourage the use of antibiotic cover for *Legionella* species.

## AUTHOR CONTRIBUTIONS

AS and MJM both contributed to (i) the conception or design of the work, the acquisition, analysis or interpretation of data for the work; (ii) drafting the work or reviewing it critically for important intellectual content; and (iii) gave final approval of the version to be published.

## CONFLICT OF INTEREST STATEMENT

Michael Maze is an Editorial Board member of Respirology Case Reports and a co‐author of this article. He was excluded from all editorial decision‐making related to the acceptance of this article for publication.

## ETHICS STATEMENT

The authors declare that appropriate written informed consent was obtained for the publication of this manuscript and accompanying images.

## Data Availability

All relevant data is available within the manuscript.
